# Psychische Belastung des intensivmedizinischen Personals in Deutschland im Verlauf der COVID-19-Pandemie. Evidenz aus der VOICE-Studie

**DOI:** 10.1007/s00063-024-01164-6

**Published:** 2024-08-07

**Authors:** Alexander Niecke, Michaela Henning, Martin Hellmich, Yesim Erim, Eva Morawa, Petra Beschoner, Lucia Jerg-Bretzke, Franziska Geiser, Andreas M. Baranowski, Kerstin Weidner, Sabine Mogwitz, Christian Albus

**Affiliations:** 1 Klinik und Poliklinik für Psychosomatik und Psychotherapie, Medizinische Fakultät und Uniklinik, Universität zu Köln, Weyertal 76, 50937 Köln, Deutschland; 2 Institut für Medizinische Statistik und Bioinformatik, Medizinische Fakultät, Universität zu Köln, Köln, Deutschland; 3 Psychosomatische und Psychotherapeutische Abteilung, Universitätsklinikum Erlangen, Friedrich-Alexander-Universität Erlangen-Nürnberg (FAU), Erlangen-Nürnberg, Deutschland; 4 Klinik für Psychosomatische Medizin und Psychotherapie, Universitätsklinikum Ulm, Ulm, Deutschland; 5 Klinik für Psychosomatische Medizin und Psychotherapie, Universitätsklinikum Bonn, Universität Bonn, Bonn, Deutschland; 6 Klinik für Psychotherapie und Psychosomatische Medizin, Medizinische Fakultät, Technische Universität Dresden, Dresden, Deutschland

**Keywords:** Intensivmedizin, Infektionskrankheit, Gesundheitspersonal, Psychische Gesundheit, PHQ‑4, Intensive care medicine, Infectious disease, Healthcare workers, Mental health, PHQ-4

## Abstract

**Hintergrund:**

Die Coronavirus(COVID-19)-Pandemie hat das Gesundheitswesen weltweit vor große Herausforderungen gestellt und zu besonderen Belastungen des medizinisch tätigen Personals geführt. Das Ziel dieser Analyse war es zu untersuchen, wie hoch in Deutschland die globale psychische Belastung von in der direkten Krankenversorgung tätigem Personal im Verlauf der COVID-19-Pandemie war.

**Methoden:**

In dieser prospektiven Querschnittstudie mit vier Messzeitpunkten (T1: 4–5/2020, T2: 11/2020–1/2021, T3: 5–7/2021, T4: 2–5/2022) wurden psychische Belastungssymptome im Rahmen eines Online-Surveys mit dem Patient Health Questionnaire (PHQ-4) bei Krankenhauspersonal, das in der direkten Patientenversorgung tätig war, erfasst (*N* = 5408 Datensätze). Der Gesamtdatensatz wurde explorativ nach Tätigkeitsbereich, Geschlecht und Berufsgruppenzugehörigkeit analysiert sowie der Verlauf über die vier Messzeitpunkte hinweg betrachtet.

**Ergebnisse:**

Eine klinisch relevante psychische Belastung (PHQ-4 ≥ 5) lag bei 29,3 % (*n* = 419/1429) des intensivmedizinischen Personals vor. Im Vergleich der vier Querschnittserhebungen zeigte sich eine signifikante Steigerung der Rate klinisch relevanter psychischer Belastung im ersten Pandemiejahr (23,2 % zu T1 vs. 30,6 % zu T2; *p* < 0,01), welche sich im zweiten Pandemiejahr auf hohem Niveau stabilisierte (33,6 % zu T3 bzw. 32,0 % zu T4). Frauen unterschieden sich dabei nicht von den Männern (*n* = 280/919 vs. *n* = 139/508 bzw. 30,4 % vs. 27,4 %; *p* = 0,74). Pflegekräfte waren signifikant häufiger psychisch belastet als ärztliches Personal (*n* = 339/1105 vs. *n* = 80/324 bzw. 30,7 % vs. 24,7 %; *p* = 0,03). Das Intensivpersonal wies keine signifikant höhere Belastung auf als das in nichtintensivmedizinischen Funktionsbereichen tätige Personal (*n* = 419/1429 vs. *n* = 1149/3979 bzw. 29,3 % vs. 28,7 %, *p* = 0,21).

**Schlussfolgerung:**

Mitarbeitende im deutschen Gesundheitswesen gaben in der Pandemie eine hohe und im Verlauf ansteigende psychische Belastung an, jedoch fand sich in unserer Stichprobe kein signifikanter Unterschied zwischen intensivmedizinischem und nichtintensivmedizinischem Personal. Dies ist möglicherweise dadurch bedingt, dass die Pandemie in Deutschland international vergleichsweise moderat verlief und weder ein Kollaps des Gesundheitssystems im Allgemeinen noch eine Dekompensation des intensivmedizinischen Versorgungssektors im Besonderen stattfand.

## Abstract (en)

**Background:**

The coronavirus disease 2019 (COVID-19) pandemic posed major challenges to the healthcare system worldwide and led to particular stress among healthcare workers. The aim of this analysis was to investigate the level of global mental stress of direct healthcare workers in Germany during the COVID-19 pandemic.

**Methods:**

In this prospective cross-sectional study with four measurement points (T1: 4–5/2020, T2:11/2020–1/2021, 5–7/2021, 2–5/2022), psychological distress symptoms were recorded in an online survey with the Patient Health Questionnaire (PHQ-4) among hospital staff working in direct patient care (*N* = 5408 datasets). The total dataset was exploratively analyzed according to field of activity, gender, and professional group affiliation.

**Results:**

Clinically relevant psychological distress (PHQ-4 ≥ 5) was present in 29.3% (*n* = 419/1429) of intensive care staff. A comparison of the four cross-sectional surveys showed a significant increase in the rate of clinically relevant mental distress in the first pandemic year (23.2% at T1 vs. 30.6% at T2; *p* < 0.01), which stabilized at a high level in the second pandemic year (33.6% at T3 and 32.0% at T4). Women did not differ from men in this respect (*n* = 280/919, 30.4% vs. *n* = 139/508, 27.4%; *p* = 0.74). Nursing staff were significantly more often psychologically stressed than physicians (*n* = 339/1105, 30.7% vs. *n* = 80/324, 24.7%; *p* = 0.03). Intensive care staff did not show significantly higher stress than staff working in nonintensive care areas (*n* = 419/1429, 29.3% vs. *n* = 1149/3979, 28.7%, *p* = 0.21).

**Conclusion:**

German healthcare workers reported high levels of mental distress during the pandemic, which increased during the course of the pandemic, but no significant difference was found between intensive care and nonintensive care staff in our sample. This may be due to the fact that the pandemic in Germany was comparatively moderate internationally and neither a collapse of the healthcare system in general nor a collapse of intensive care structures in particular took place.

## Hintergrund

Die Coronavirus(COVID-19)-Pandemie hat seit Anfang 2020 weltweit das medizinische Personal vor große Herausforderungen gestellt. In den Kliniken kam es zu einer hohen Zahl stationär behandlungsbedürftiger Fälle von mit SARS-CoV‑2 infizierten Personen, oftmals mit komplexen intensivpflichtigen Verläufen und Todesfällen relativ junger Menschen. Das klinisch tätige Personal war während der COVID-19-Pandemie weit überdurchschnittlichen Belastungen ausgesetzt, insbesondere durch ein erhöhtes Infektionsrisiko und lange Arbeitszeiten aufgrund knapper Personalressourcen. Am Anfang der Pandemie mangelte es in Deutschland zudem an Masken und Schutzkleidung sowie an einer Schutzimpfung [1]. In einem Umbrella-Review aus dem Jahr 2022 der psychischen Gesundheit von Fachkräften im Gesundheitswesen während der COVID-19-Pandemie wurden weltweit Ängste, Depressivität und posttraumatische Belastungssymptome als häufige pandemiebedingte psychische Belastungen identifiziert [2] mit Prävalenzraten von jeweils ca. 20–35 % für Depressionen bzw. Angststörungen [3, 4]. Pflegefachpersonen wiesen dabei höhere Belastungswerte auf als ärztlich Beschäftigte [5–8]. Risikofaktoren für eine reduzierte psychische Gesundheit beim Gesundheitspersonal waren das weibliche Geschlecht [2, 9, 10], jüngeres Lebensalter [2, 10], Kontakt mit Infizierten [2], Angst vor Infektion, häusliches Zusammenleben mit älteren Menschen [11] und Überforderungserleben [12]. Als protektive Faktoren wurden demgegenüber Wertschätzung und emotionale Unterstützung im Team sowie das Erleben von Selbstwirksamkeit benannt [13].

Im Vergleich mit dem Personal anderer medizinischer Funktionsbereiche war im Intensivbereich die Rate an berufsbezogener emotionaler Erschöpfung („Burn-out-Syndrom“) in Deutschland bereits präpandemisch erhöht [14], und von den Mitgliedern der beiden mitgliederstärksten deutschen intensivmedizinischen Fachgesellschaften wurde Bedarf an psychosozialer Unterstützung ihrer Teams angezeigt [15, 16]. Während der COVID-19-Pandemie wurden von Pflegefachpersonen körperliche Belastungen wie das lange Arbeiten in kompletter Schutzkleidung oder Lagerungen von häufig adipösen Personen sowie emotionale Belastungen durch eine hohe Sterblichkeitsrate und die Notwendigkeit der Sterbebegleitung als besondere Herausforderungen beschrieben [17]. Ärztinnen und Ärzte empfanden demgegenüber Triage-Entscheidungen, insbesondere durch Ablehnungen aus Kapazitätsgründen, als belastend [17]. Einige internationale Studien zeigen bei Intensivpersonal unter Pandemiebedingungen hohe psychische Belastungswerte [18–20]. Es existieren Hinweise, dass das Gesundheitspersonal in Deutschland gut aufgeklärt war und das Gesundheitssystem aufgrund eines moderaten Pandemieverlaufs mit vergleichsweise niedriger Sterberate – anders als in Ländern wie China, USA, Brasilien, Italien und Spanien – nicht über die Grenzen beansprucht wurde, sodass das Gesundheitspersonal hier insgesamt weniger belastet erschien [21].

Die Zielsetzung der vorliegenden Analyse war (1) die Identifikation von klinisch relevanter psychischer Belastung des intensivmedizinischen Personals in Deutschland im Quer- und Längsschnitt, (2) die Analyse von Berufsgruppen- und Geschlechterunterschieden und (3) ein Vergleich des intensivmedizinisch vs. nichtintensivmedizinisch tätigen Personals.

## Methodik

### Studiendesign

Bei der VOICE-Studie handelt es sich um eine prospektive Beobachtungsstudie von Beschäftigten im Gesundheitswesen mit vier Messzeitpunkten während der COVID-19-Pandemie. Die vorliegende Studie wurde von allen Ethikkommissionen der fünf teilnehmenden Universitätskliniken (Bonn/BN, Nürnberg-Erlangen/N-ER, Dresden/DD, Köln/K und Ulm/U) genehmigt und auf ClinicalTrials registriert (DRKS-ID: DRKS00021268). Alle Befragten gaben online eine schriftliche Einwilligungserklärung zur Studienteilnahme ab.

### Datenerhebung

An vier deutschen Universitätskliniken (BN, DD, K, U) wurde das via Mailinglisten erreichbare Klinikpersonal personalisiert angeschrieben und über den Survey informiert; in N‑ER wurde über das Mitarbeiterportal auf die Studie aufmerksam gemacht. Außerdem wurden Mitarbeitende diverser anderer Kliniken via Social Media (z. B. Medscape) und über Fachverbände/-gesellschaften adressiert. Die Erhebung erfolgte zu vier Messzeitpunkten mittels anonymisierter Onlinefragebögen (T1: April bis Mai 2020; T2: November 2020 bis Januar 2021; T3: Mai bis Juli 2021; T4: Februar bis Mai 2022). Die Messzeitpunkte zu T1, T2 und T4 entsprachen jeweils dem Bereich eines Infektionsgipfels („Welle“), wobei zu T3 fallende Infektionszahlen vorlagen. Die insgesamt 15- bis 20-minütige Gesamtumfrage wurde mit zwei akademischen Online-Umfragetools programmiert: Unipark (www.unipark.com) und SoSci Survey (www.soscisurvey.de). Einschlusskriterien waren ein Mindestalter von 18 Jahren, Tätigkeit im Gesundheitswesen sowie ein Arbeitsplatz in Deutschland. Zu jedem Messzeitpunkt was es möglich, mit der Teilnahme zu beginnen.

### Datenanalyse

In der vorliegenden Analyse wurden aus dem Gesamtdatensatz nur Teilnehmende, die in deutschen Krankenhäusern ärztlich oder pflegerisch in somatischen Fächern mit direkter Patientenversorgung beschäftigt waren und den PHQ-4-Fragebogen vollständig ausgefüllt haben, berücksichtigt. Für die Beschreibung der Gesamtbelastung wurden alle Datensätze gepoolt, um eine möglichst große statistische Power zu erreichen. Für die Verlaufsuntersuchung wurden die Querschnittserhebungen einzeln verglichen und der longitudinalen Untersuchung gegenübergestellt.

### Soziodemografische Variablen

Es wurden folgende Merkmale untersucht: Geschlecht (männlich vs. weiblich), Berufsgruppe (ärztlich vs. pflegerisch) und Tätigkeitsbereich (Intensivstation vs. sonstige in der somatischen Krankenversorgung angegliederte Stationen).

### Psychometrische Variablen

Die Ultrakurzform des Gesundheitsbogens für Patienten (Patient Health Questionnaire‑4 [PHQ-4]; dt. Version 2015; [22]) ist ein dimensionales Selbstbeurteilungsinstrument mit vier Items zur Erfassung von Angst und Depressivität. Der PHQ‑4 beinhaltet zwei Subskalen, die jeweils die diagnostischen Kernkriterien der „major depression“ (PHQ-2) bzw. der generalisierten Angststörung (GAD-2) erfassen. Als Maß der generellen psychischen Belastung kann der PHQ-4-Gesamtscore verwendet werden, der Summenwerte von 0 bis 12 erreichen kann. Dabei ist ein Cut-off-Wert von ≥ 5 ein Marker für wahrscheinliche Fälle von klinisch relevanten depressiven und/oder Angstsymptomen. In der vorliegenden Analyse wurde dieser Cut-off als primäres Zielkriterium gewählt. Die Reliabilität, konvergente, divergente und faktorielle Validität des PHQ‑4 können als gut bezeichnet werden (Cronbachs α = 0,85). Der mittlere Kennwert beträgt 2,5 (SD = 2,8) für US-amerikanische Hausarztklientel [23].

### Statistische Analyse

Die statistische Analyse der soziodemografischen, psychometrischen und pandemiebezogenen Merkmale erfolgte deskriptiv mittels absoluter und relativer Häufigkeiten (Kreuztabulation); zudem wurden inferenzstatistische Vergleiche von Anteilen auf der Basis von verallgemeinerten Schätzgleichungen (wegen der wiederholten Befragungen über die Zeit) gemacht (engl. *„generalized estimating equations“ *[GEE]). Die Berechnungen wurden in SPSS Statistics, Version 28 (IBM Corp., Armonk, NY, USA), durchgeführt. Die präsentierten *p*-Werte sind unadjustiert, für eine Multiplizitätskorrektur könnten alle *p*-Werte mit 29 multipliziert werden (Bonferroni-Korrektur).

## Ergebnisse

### Stichprobenbeschreibung

Insgesamt nahmen 19.972 Personen des Gesundheitswesens an dem Survey teil. Pro Person wurden bis zu vier Datensätze erhoben (Teilnahme: viermalig: 122, dreimalig: 498, zweimalig: 1922, einmalig: 17.430). Hieraus ergaben sich insgesamt 23.256 Datensätze (T1: 8067, T2: 7190, T3: 3463, T4: 4536), die andernorts bereits publiziert wurden (z. B. [24–26]). In der vorliegenden Subgruppenanalyse werden nur die Daten von in der direkten somatischen Patientenversorgung („am Bett“) tätigen ärztlichen und pflegerischen Berufsgruppen präsentiert (*n* = 5408 Datensätze), wobei hiervon rund ein Viertel aus dem intensivmedizinischen Kontext stammt (*n* = 1429; 26,4 %; Tab. 1).

**Tab. 1:** Anzahl der Befragten, gruppiert nach Geschlecht, Beruf, Tätigkeitsbereich und Verlauf

		Intensivstationen [*n* = 1429]				Sonst. somatische Stationen [*n* = 3979]				Total [*n* = 5408]			
	Verlauf	T1 [*n*]	T2 [*n*]	T3 [*n*]	T4 [*n*]	T1 [*n*]	T2 [*n*]	T3 [*n*]	T4 [*n*]	T1 [*n*]	T2 [*n*]	T3 [*n*]	T4 [*n*]
Total	–	409	467	250	303	1290	1356	610	723	1699	1823	860	1026
Geschlecht	Weiblich	269	279	164	209	864	928	451	525	1133	1207	615	734
	Männlich	140	188	86	94	426	428	159	198	566	616	245	292
Beruf	Ärztlich	110	112	47	55	786	787	273	341	896	899	320	396
	Pflegerisch	299	355	203	248	504	569	337	382	803	924	540	630
Beruf und Geschlecht	Ärztin	62	45	20	30	457	456	177	214	519	501	197	244
	Arzt	48	67	27	25	329	331	96	127	377	398	123	152
	Pflegefachfrau	207	234	144	179	407	472	274	311	614	706	418	490
	Pflegefachmann	92	121	59	69	97	97	63	71	189	218	122	140

### Ausmaß der globalen psychischen Belastung

Wie in Tab. 2 dargestellt, gab fast ein Drittel des klinisch tätigen Gesundheitspersonals in der Stichprobe während des zweijährigen Gesamterhebungszeitraums eine globale psychische Belastung von PHQ-4 ≥ 5 an (*n* = 1554/5408; 28,8 %). Das intensivmedizinische Personal unterschied sich hierbei nicht von dem nichtintensivmedizinisch tätigen Personal (*n* = 419/1429 vs. *n* = 1149/3979 bzw. 29,3 % vs. 28,7 %; *p* = 0,21; Tab. 2).

**Tab. 2:** Relative Häufigkeiten psychischer Belastung (PHQ-4≥5), gruppiert nach Geschlecht, Beruf und Tätigkeitsbereich, T1–T4 (*n* = 5408)

		Intensivstationen (IST)				Sonstige somatische Stationen (SOM)				IST vs. SOM	Total			
		*n*	PHQ+ [*n*]	PHQ+ [%]	*p*‑Wert	*n*	PHQ+ [*n*]	PHQ+ [%]	*p*‑Wert	*p*-Wert	*n*	PHQ+ [*n*]	PHQ+ [%]	*p*-Wert
Total	–	1429	419	29,3	–	3979	1140	28,7	–	0,214	5408	1559	28,8	–
Geschlecht	Weiblich	919	280	30,4	0,738	2768	815	29,4	0,877	0,490	3689	1095	29,7	0,828
	Männlich	508	139	27,4		1211	325	26,8		0,298	1719	464	27,0	
Beruf	Ärztlich	324	80	24,7	0,030	2187	565	25,8	< 0,001	0,814	2511	645	25,7	< 0,001
	Pflegerisch	1105	339	30,7		1792	575	32,1		0,074	2897	914	31,5	
Beruf und Geschlecht	Ärztin	157	38	24,2	0,848	1304	362	27,8	0,016	0,331	1461	400	27,4	0,481
	Arzt	167	42	25,1		883	203	23,0		0,576	1050	245	23,3	
	Pflegefachfrau	764	242	31,7	0,313	1464	453	30,9	0,042	0,733	2228	695	31,2	0,544
	Pflegefachmann	341	97	28,4		328	122	37,2		0,024	669	219	32,7	

PHQ+ = PHQ-4 ≥ 5

### Vergleich der berufsgruppen- und geschlechtsbezogenen Merkmale

Im Berufsgruppenvergleich zeigten sich im intensivmedizinischen Kontext Pflegefachpersonen signifikant höher belastet als ihre ärztlichen Kolleginnen und Kollegen (*n* = 339/1105 vs. *n* = 80/324 bzw. 30,7 % vs. 24,7 %; *p* = 0,03), wobei dieser Unterschied auf den nichtintensivmedizinischen Stationen noch deutlicher ausfiel (*n* = 565/2187 vs. *n* = 575/1792 bzw. 32,1 % vs. 25,8 %; *p* < 0,001).

Im Geschlechtervergleich unterschieden sich intensivmedizinisch tätige Frauen hinsichtlich der Rate psychisch Belasteter nicht gegenüber ihren männlichen Kollegen (*n* = 280/919 vs. *n* = 139/508 bzw. 30,4 % vs. 27,4 %, *p* = 0,74). Auf den peripheren somatischen Stationen zeigte sich beim Geschlechtervergleich eine analoge Konstellation. Für die Vergleichsanalysen wurden Befragte mit nonbinärer Geschlechtsangabe (*n* = 17; 0,003 % der Gesamtpopulation) nicht berücksichtigt, die Rate psychischer Belastung lag in dieser Gruppe bei 41,2 % (*n* = 7/17). Bei weiterer Differenzierung mit Kombination der berufsgruppen- und geschlechtsbezogenen Merkmale zeigte sich, dass männliche Pflegefachpersonen im intensivmedizinischen Bereich signifikant weniger belastet waren als ihre geschlechtsgleichen Kollegen im nichtintensivmedizinischen Bereich (*n* = 97/341 vs. 122/328 bzw. 28,4 % vs. 37,2 %, *p* < 0,02).

### Veränderung der psychischen Belastung im Pandemieverlauf

Im Verlauf der COVID-19-Pandemie stieg die globale psychische Belastung von Frühjahr 2020 (T1) bis Winter 2020/2021 (T2) signifikant an (Gesamtstichprobe: *n* = 394/1699 vs. *n* = 585/1823 bzw. 23,2 % vs. 32,1 %, *p* < 0,01) und stabilisierte sich anschließend mit nichtsignifikanten Schwankungen bei den Messungen im zweiten Pandemiejahr auf hohem Niveau (T3: *n* = 250/860; 29,2 %; *p* = 0,13; T4: *n* = 329/1026; 32,1 %; *p* = 0,16) – dieser Effekt bestätigte sich bei der longitudinalen Auswertung (*n* = 122 Befragte, die an allen vier Messzeitpunkten teilnahmen). Während sich zu T1 die Rate relevanter klinischer Belastung von intensivmedizinisch und nichtintensiv tätigem Personal nicht unterschied (*n* = 95/409 vs. *n* = 299/1290 bzw. jeweils 23,2 %), war zu T2 das nichtintensivmedizinisch tätige Personal (*n* = 437/1356; 32,2 %) und zu T3 sowie zu T4 das Intensivpersonal stärker belastet (*n* = 84/250; 33,6 % bzw. *n* = 97/303 bzw. 32,0 %). Eine Auffälligkeit zeigte sich im Sommer 2021 (T3): Während ärztlich Beschäftigte, insbesondere auf den Intensivstationen, die niedrigsten Belastungswerte aufwiesen, zeigten Pflegefachpersonen die höchsten Belastungswerte (Tab. 3).

**Tab. 3:** Relative Häufigkeiten psychischer Belastung, modelliert, gruppiert nach Tätigkeitsbereich im Vergleich der vier Querschnittsuntersuchungen (T1-T4)

	T1 [%]	T2 [%]	*p* [T1-T2]	T3 [%]	*p* [T2-T3]	T4 [%]	*p* [T3-T4]
*Intensivmedizin*	23,2	30,6	< 0,001	33,6	0,59	32,0	0,67
Ärztlich	21,6	26,8		17,0		32,7	
Pflegerisch	23,7	32,0		37,4		31,9	
*Nichtintensivmedizin*	23,2	32,7	< 0,001	27,4	0,045	32,2	0,09
Ärztlich	21,4	30,3		21,2		29,3	
Pflegerisch	25,9	35,9		32,4		34,7	
*Total*	23,2	32,1	< 0,001	29,2	0,64	32,1	0,66
Ärztlich	21,5	29,8		20,6		29,8	
Pflegerisch	25,1	34,4		34,3		33,6	

## Diskussion

### Hauptergebnis

Im Hauptergebnis zeigte sich, dass sich während der COVID-19-Pandemie die Rate des psychisch belasteten Personals im intensivmedizinischen vs. nichtintensivmedizinischen Kontext nicht signifikant voneinander unterschied. Hinsichtlich des Verlaufs war im zweiten Pandemiejahr die psychische Belastungsrate höher als zu Beginn der Pandemie.

### Stärken und Schwächen

Als besondere Stärke der Studie können die Größe der Stichprobe und die vier sequenziellen Erhebungszeitpunkte angesehen werden. Limitierend ist zu beachten, dass keine präpandemischen Ausgangswerte und keine Kontrollgruppe aus der Allgemeinbevölkerung vorliegen. Außerdem wurde lediglich ein ultrakurzes Instrument zum Screening auf psychische Belastung verwendet. Für den PHQ‑4 konnte die Reliabilität mit Cronbachs α = 0,85 repliziert werden [23]. Auch sind Selektionseffekte durch das Studiendesign (z. B. Online-Survey) anzunehmen. So war die Anzahl des befragten ärztlichen und pflegerischen Personals auf den nichtintensivmedizinischen Stationen zwar annähernd gleich verteilt, auf den Intensivstationen nahmen allerdings 3‑ bis 4‑fach mehr Pflegefachpersonen als ärztlich Mitarbeitende teil, sodass hier sogar eine Überschätzung der psychischen Belastung des Intensivpersonals möglich ist. Dass rund ein Viertel der Befragten aus dem intensivmedizinischen Kontext stammt, entspricht nicht der üblichen Verteilung in deutschen Krankenhäusern und verweist auf eine deutlich höhere Beteiligungsrate auf Intensivstationen als in den anderen Bereichen. Dies ist ein Hinweis, dass Mitarbeitende auf Intensivstationen das Thema psychische Belastung in der Pandemie stärker beschäftigt haben könnte, und stellt gleichzeitig ein Problem für die Generalisierbarkeit des Vergleichs dar. Eine weitere Limitation ist, dass die Probanden fast ausschließlich aus dem Bereich der universitären Maximalversorgung kamen und somit die Studienergebnisse nicht als repräsentativ für die gesamte intensivmedizinische Versorgungslandschaft in Deutschland gelten kann.

### Interpretation und Vergleich mit der Literatur

Eine vorherige Analyse der VOICE-Daten konnte bereits zeigen, dass die Belastungswerte von Klinikpersonal im Vergleich zu den normativen Werten der deutschen Allgemeinbevölkerung generell deutlich erhöht waren [24]. Anders als in anderen Publikationen beschrieben [18–20], zeigte sich in der vorliegenden Untersuchung allerdings kein signifikanter Unterschied zwischen dem intensivmedizinischen und dem nichtintensivmedizinischen Personal. Dieser Befund könnte dadurch erklärt werden, dass intensivmedizinisches Personal trotz der besonderen Herausforderungen auch positive Erfahrungen erlebt hat, beispielsweise durch Zusammenhalt im Team und solidarische Unterstützung von außen [17]. Auch eine hohe mediale Aufmerksamkeit mit wertschätzender Berichterstattung mag dazu beigetragen haben, dass Selbstwirksamkeit und Sinnhaftigkeit erlebt werden konnten und für mentale Entlastung gesorgt haben [17]. Nicht zuletzt können regionale Faktoren, wie ein im internationalen Vergleich in Deutschland relativ moderater Pandemieverlauf bei einer weitestgehend kompensierten und hochfunktionalen intensivmedizinischen Infrastruktur, einen entscheidenden protektiven Einfluss gehabt haben [21].

Das in der Literatur mehrfach beschriebene Phänomen einer höheren psychischen Belastung des Pflegepersonals gegenüber dem ärztlichen Personal [5–8] konnten wir auch im intensivmedizinischen Kontext replizieren. Allerdings konnten wir das Phänomen einer höheren psychischen Belastung beim weiblichen gegenüber dem männlichen Geschlecht [2, 9, 10] nicht nachweisen.

Im zeitlichen Verlauf stieg die Rate psychisch belasteter Personen mit der zweiten Infektionswelle (Winter 2020/21) gegenüber der ersten Infektionswelle (Frühjahr 2020) signifikant an und blieb bei den weiteren Erhebungen auf relativ hohem Niveau. Der signifikante Anstieg zu T2 korreliert dabei mit der Tatsache, dass es im Winter 2020/21 in Deutschland einerseits die bislang höchste Anzahl an mit COVID-19 Infizierten belegten Intensivbetten und COVID-19-bedingten Todesfällen gab und andererseits immer noch eine flächenweit verfügbare Möglichkeit zur Schutzimpfung fehlte (www.intensivregister.de/#/index). Auch wenn es im Sommer 2021 (T3) nach der dritten Welle zu einer deutlichen Reduktion der intensivpflichtigen COVID-19-Verläufe kam, eine Schutzimpfung verfügbar war und oftmals psychosoziale Unterstützungsangebote etabliert waren, zeigte sich keine Erholung des intensivmedizinischen Personals, insbesondere der Intensivpflege. Im Gegenteil wies das intensivmedizinische Personal zu T3 mit 33,6 % die höchste Belastungsrate im Untersuchungsintervall auf, wobei hierbei eine sich verschärfende Personalnot eine entscheidende Rolle gespielt haben mag.

## Ausblick

Zukünftige Forschungsarbeiten sollten die psychische Gesundheit anhand repräsentativer Stichproben mit Studiendesigns, die elaboriertere Diagnostiktools beinhalten, regelmäßig überprüfen, um rechtzeitig geeignete Maßnahmen treffen zu können, um die Gesundheit und die Arbeitsfähigkeit dieser Schlüsselmitglieder der öffentlichen Gesundheit aufrechtzuerhalten. Darüber hinaus ist zu bedenken, dass die Aufmerksamkeit in unserer Gesellschaft mittlerweile weitergewandert ist, aber pandemisch bedingte psychische Belastungen auch postpandemisch länger nachwirken können und kumulativ eher ansteigen. Eine Entlastung und Unterstützung ist deshalb nicht nur in einer Krise, sondern insbesondere nach einer Krise erforderlich und geboten.

## Fazit für die Praxis

- Das in der direkten Krankenversorgung tätige Gesundheitspersonal war während der COVID-19-Pandemie psychisch hoch belastet; im zweiten Pandemiejahr war die psychische Belastung stärker als zu Beginn der Pandemie.
- Im Unterschied zu anderen Ländern war in Deutschland das intensivmedizinische Personal nicht stärker belastet als das nichtintensivmedizinische Personal.
- Auf Intensivstationen waren Pflegefachpersonen jedoch stärker belastet als das ärztliche Personal.
