# Erratum zu: Psychische Belastung des intensivmedizinischen Personals in Deutschland im Verlauf der COVID-19-Pandemie. Evidenz aus der VOICE-Studie

**DOI:** 10.1007/s00063-025-01255-y

**Published:** 2025-02-07

**Authors:** Alexander Niecke, Michaela Henning, Martin Hellmich, Yesim Erim, Eva Morawa, Petra Beschoner, Lucia Jerg-Bretzke, Franziska Geiser, Andreas M. Baranowski, Kerstin Weidner, Sabine Mogwitz, Christian Albus

**Affiliations:** 1 Klinik und Poliklinik für Psychosomatik und Psychotherapie, Medizinische Fakultät und Uniklinik, Universität zu Köln, Weyertal 76, 50937 Köln, Deutschland; 2 Institut für Medizinische Statistik und Bioinformatik, Medizinische Fakultät, Universität zu Köln, Köln, Deutschland; 3 Psychosomatische und Psychotherapeutische Abteilung, Universitätsklinikum Erlangen, Friedrich-Alexander-Universität Erlangen-Nürnberg (FAU), Erlangen-Nürnberg, Deutschland; 4 Klinik für Psychosomatische Medizin und Psychotherapie, Universitätsklinikum Ulm, Ulm, Deutschland; 5 Klinik für Psychosomatische Medizin und Psychotherapie, Universitätsklinikum Bonn, Universität Bonn, Bonn, Deutschland; 6 Klinik für Psychotherapie und Psychosomatische Medizin, Medizinische Fakultät, Technische Universität Dresden, Dresden, Deutschland

**Erratum zu:**

**Med Klin Intensivmed Notfmed 2025**

10.1007/s00063-024-01164-6

Der Artikel „Psychische Belastung des intensivmedizinischen Personals in Deutschland im Verlauf der COVID-19-Pandemie. Evidenz aus der VOICE-Studie“, geschrieben von Alexander Niecke, Michaela Henning, Martin Hellmich, Yesim Erim, Eva Morawa, Petra Beschoner, Lucia Jerg-Bretzke, Franziska Geiser, Andreas M. Baranowski, Kerstin Weidner, Sabine Mogwitz und Christian Albus, wurde ursprünglich unter exklusiver Lizenz von Springer Medizin Verlag GmbH, ein Teil von Springer Nature 2024 veröffentlicht. Als Ergebnis der anschließenden Entscheidung, den Artikel im Open-Access-Modell zu veröffentlichen, wurde der Copyright-Vermerk des Artikels am 3. Januar 2025 in © The Author(s) 2024 geändert und der Artikel wird nun unter der Creative Commons Namensnennung 4.0 International-Lizenz veröffentlicht, welche die Nutzung, Vervielfältigung, Bearbeitung, Verbreitung und Wiedergabe in jeglichem Medium und Format erlaubt, sofern Sie den/die ursprünglichen Autor(en) und die Quelle ordnungsgemäß nennen, einen Link zur Creative-Commons-Lizenz beifügen und angeben, ob Änderungen vorgenommen wurden.

Die in diesem Artikel enthaltenen Bilder und sonstiges Drittmaterial unterliegen ebenfalls der genannten Creative-Commons-Lizenz, sofern sich aus der Abbildungslegende nichts anderes ergibt. Sofern das betreffende Material nicht unter der genannten Creative-Commons-Lizenz steht und die betreffende Handlung nicht nach gesetzlichen Vorschriften erlaubt ist, ist für die oben aufgeführten Weiterverwendungen des Materials die Einwilligung des jeweiligen Rechteinhabers einzuholen.

Weitere Details zur Lizenz entnehmen Sie bitte der Lizenzinformation auf http://creativecommons.org/licenses/by/4.0/deed.de.

Open Access gefördert durch: Project DEAL.

